# Relative abundance of oceanic juvenile loggerhead sea turtles in relation to nest production at source rookeries: implications for recruitment dynamics

**DOI:** 10.1038/s41598-019-49434-0

**Published:** 2019-09-10

**Authors:** Frederic Vandeperre, Hugo Parra, Christopher K. Pham, Miguel Machete, Marco Santos, Karen A. Bjorndal, Alan B. Bolten

**Affiliations:** 1 IMAR – Institute of Marine Research, Departamento de Oceanografia e Pescas, Universidade dos Açores, Horta, Portugal; 2 MARE – Marine and Environmental Sciences Centre, Departamento de Oceanografia e Pescas, Universidade dos Açores, Horta, Portugal; 3 OKEANOS – Departamento de Oceanografia e Pescas, Universidade dos Açores, Horta, Portugal; 4 DRAM – Direção Regional dos Assuntos do Mar, Direção de Serviços de Biodiversidade e Politica do Mar, Horta, Portugal; 5 ACCSTR – Archie Carr Center for Sea Turtle Research, University of Florida, Gainesville, FL USA

**Keywords:** Marine biology, Population dynamics

## Abstract

After hatching, juveniles of most sea turtle species undertake long migrations across ocean basins and remain in oceanic habitats for several years. Assessing population abundance and demographic parameters during this oceanic stage is challenging. Two long-recognized deficiencies in population assessment are (i) reliance on trends in numbers of nests or reproductive females at nesting beaches and (ii) ignorance of factors regulating recruitment to the early oceanic stage. To address these critical gaps, we examined 15 years of standardized loggerhead sighting data collected opportunistically by fisheries observers in the Azores archipelago. From 2001 to 2015, 429 loggerheads were sighted during 67,922 km of survey effort. We used a model-based approach to evaluate the influence of environmental factors and present the first estimates of relative abundance of oceanic-stage juvenile sea turtles. During this period, relative abundance of loggerheads in the Azores tracked annual nest abundance at source rookeries in Florida when adjusted for a 3-year lag. This concurrence of abundance patterns indicates that recruitment to the oceanic stage is more dependent on nest abundance at source rookeries than on stochastic processes derived from short term climatic variability, as previously believed.

## Introduction

Knowledge of a species’ abundance and demography is essential for population assessment and designing effective conservation strategies. However, understanding demographic processes is challenging, especially for species with complex life histories that involve large scale migrations (e.g. tuna^1^; sharks^2,3^; whales^4^; seabirds^5^; and sea turtles^6^). The loggerhead sea turtle (*Caretta caretta*) is a good example of a highly migratory species with a complex life cycle characterized by successive ontogenetic habitat shifts from terrestrial habitats where nesting and embryonic development occur to developmental and foraging habitats in the open ocean and, later, in neritic waters^7–10^. Loggerhead sea turtles are distributed throughout tropical and temperate regions worldwide and, for management purposes, are subdivided into nine distinct population segments (DPS), which are classified as either threatened or endangered^11^. Age at sexual maturity for males and females is about 36 to 42 years^12^.

Of all loggerhead sea turtle life stages, the post-hatchling and oceanic juvenile stages are the least understood^8^. Oceanic-stage juveniles generally occur in low densities over vast oceanic areas^9,13^, which strongly hampers their study. As a consequence, the abundance and demography of oceanic-stage juveniles are poorly known, and population models continue to rely heavily on a set of assumptions regarding critical parameters (e.g. stage-specific survival rates, for details^14^). Trends in abundance of sea turtle populations have relied almost entirely on counts of nesting females and/or the nests they deposit^10^. In the Northwest Atlantic loggerhead population, sexual maturity is reached after 36–38 years in females^12^. Thus, any changes in abundance in juvenile stages as a result of new sources or intensities of mortality such as from marine pollution and fisheries^15,16^, will not be recognized until that age class reaches sexual maturity and arrives at the nesting beach. The importance of monitoring population trends of oceanic-stage juveniles as part of an “early warning system” – as we do for the first time in this paper – has been identified as a high priority^10,17–20^.

A key life-history transition is that of recruitment from nesting beaches to the oceanic juvenile stage. This transition is thought to be associated with high mortality (19–51%^13,21–24^) and encompasses several demographic processes and potential hazards such as nest destruction, hatchling emergence success, hatchling and neonate survival, and dispersal patterns driven by ocean currents^7^. These processes could be either density-dependent or -independent and could have a large stochastic component^10^. With this complexity in mind, we considered two basic and contrasting recruitment scenarios to characterise this transition, recognizing that reality may lie somewhere in between: (i) stable recruitment, with a recruitment rate that is a constant, predictable function of nest production in source rookeries, and (ii) variable recruitment, with a recruitment rate that is highly variable, dependent on stochastic events, and therefore largely unpredictable. Under stable recruitment, nest production is the main driver determining the number of individuals that enter the oceanic juvenile stage, and fluctuations in survival throughout this transition are small. Variable recruitment suggests a transition that is determined by largely stochastic processes, which can conceivably be linked to environmentally mediated mortality and survival rates. Sweepstakes recruitment is a particular example of this latter mechanism in which a small and often changing proportion of the population successfully contributes to the replenishment of the entire population in each year under influence of environmental stochasticity^25^. Such sweepstake effect has been reported in sea urchins^26^, sardines and anchovies^27^ and may exist in West Atlantic green turtles^28^.

Contemporary population models for Northwest Atlantic loggerheads rely heavily on nest counts in the source rookeries, and assume minimal variation in annual survival rates within the oceanic realm and therefore relatively stable recruitment from oceanic to neritic habitats after approximately the first decade of life^14,29^. These assumptions have been challenged by recent modeling studies that inferred a predominantly variable recruitment determined by highly variable neonate survival under influence of climate forcing^30,31^. The lack of empirical data on the abundance and recruitment of oceanic juveniles has so far precluded assessing these contrasting hypotheses directly, despite the fundamental importance for conservation planning.

The Azores archipelago is an important feeding and developmental ground for oceanic-stage loggerhead sea turtles^9,13,32^. Genetic work has revealed that the loggerheads found here mainly belong to the Northwest Atlantic DPS and originate from the southeastern United States (90%^33,34^) namely in Florida, one of the two largest nesting aggregations of loggerheads in the world^35^. Between late June and early November each year, loggerhead hatchlings with a curved carapace length (CCL) of about 5 cm emerge from approximately 1500 km of nesting beaches from Florida to North Carolina^36^ and enter the sea to be dispersed into the North Atlantic Gyre by the Gulf Stream^9,32^. In the Azores, recorded sizes of loggerhead sea turtles range from 8.5 to 82 cm CCL^13^ and the duration of the oceanic juvenile phase is estimated to average between 9 to 12 years^37,38^. These juveniles are primarily epipelagic^9^ and appear to actively explore the vicinity of seamounts and peaks^39^, which are common features in the region^40^.

Since 2001, the Azores Fishery Observer Program (POPA - Programa de Observação das Pescas dos Açores) has collected sighting data under a standardised protocol to document the occurrence of loggerhead sea turtles during operations of the pole and line tuna fishery. Based on these data, the objectives of the present study were (1) to present the first time series of relative abundance of oceanic juvenile stage loggerhead sea turtles in the North Atlantic; and (2) to assess the correlation with contemporary nest counts from the major source rookeries to investigate whether recruitment to the oceanic juvenile stage is stable or variable. The results will allow us to evaluate the use of opportunistic platforms to monitor low density aggregations such as oceanic juvenile stage loggerheads.

## Methods

### Study area

The Azores archipelago comprises nine volcanic islands divided into three groups (eastern, central and western) separated by deep waters (>2000 m). Shallow waters (<600 m) cover <1% of the almost 1 million km^2^ of the Azorean EEZ. The region is largely dominated by two eastward flows originating from the Gulf Stream: the cold North Atlantic Current in the North, and the warm Azores Front/Current system to the South. Mean sea surface temperature varies between 15 to 20 °C in winter and 20 to 25 °C in summer.

### Survey design

The data were collected under the Azorean Fisheries Observer Program (POPA; www.popaobserver.org) and consisted of dedicated visual transects for sea turtles, which were performed following a fixed protocol by fishery observers on-board pole-and-line tuna vessels^41^. The pole-and-line tuna fleet operated in the Azores between May and November and was subject to observer coverage of 50–100%. The data consisted of 15-minute visual transects which were performed up to 6 times a day (every 2 hours, i.e. at 09:00, 11:00, 13:00, 15:00, 17:00 and 19:00). Transects had no predetermined track and were only performed when the vessels were travelling or searching for tuna, and when weather conditions were favourable (i.e. sea state < 6 Beaufort). Sea turtle sightings were recorded from the flying bridge, approximately 8 m above sea level, on both sides of the vessel. Observers were equipped with Bushnell Marine binoculars (7 × 50) with integrated compass and a GPS device. Sighting angle (°) was calculated from the recorded compass heading of the vessel (°N) and the sighting (°N), while distance (m) from the vessel was estimated visually. At the beginning and at the end of each transect, observers recorded geographic location, time, sea and weather conditions (sea state – Beaufort scale, glare and visibility). Weather conditions were estimated using a categorical scale following standard POPA procedures. All observers received an intensive 10-day training before being deployed.

### Data filtering

**Figure 1:**
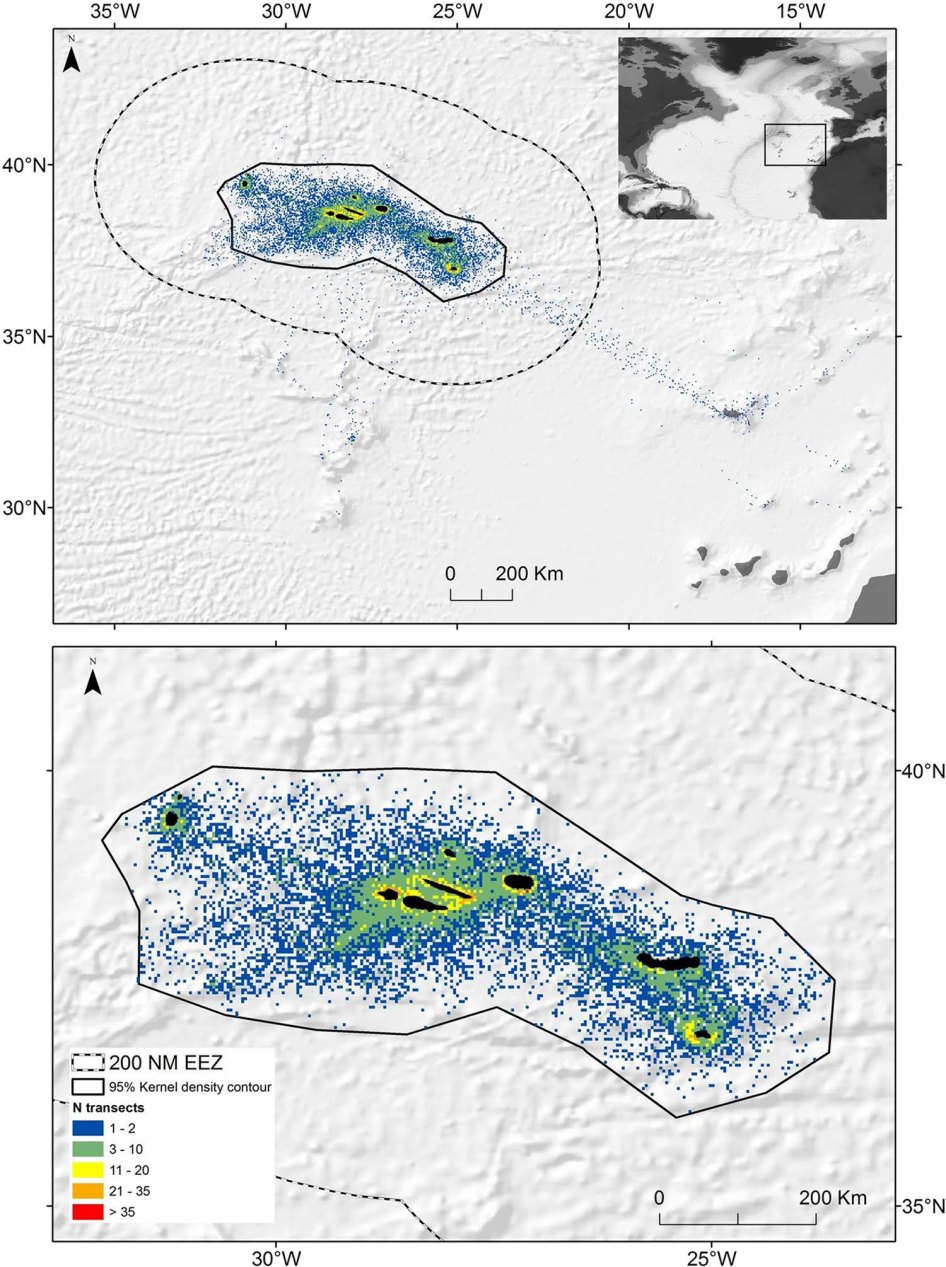
Map of the study area showing locations of the standardised line transects performed between 2001 and 2015 by fishery observers from the Azores Fishery Observer Program (POPA - Programa de Observação das Pescas dos Açores) onboard pole-and-line tuna vessels before (top panel) and after (bottom panel) filtering. The black line shows the 95% Kernel area contour for the filtered data. The dashed line shows the 200 nautical mile Exclusive Economic Zone.

The database covered the period from 2001 to 2015 and contained 22,341 transects (Fig. 1) and 515 turtle sightings. The great amount of data generated by this methodology is counterbalanced by one obvious limitation: the lack of experimental design inherent to this opportunistic methodology results in an unbalanced spatial and temporal coverage that is driven by the fishing activity. The data were cleaned and subsequently filtered in order to reduce the effect of the sampling bias using the following steps: 1) transects were filtered using the distance between the beginning and end point to obtain credible, semi-linear transects with lengths ranging between 1.35 and 5.40 km, corresponding to average vessel speeds between 5.40 and 21.60 km h^−1^, respectively; 2) transects conducted with sea conditions above 3 Beaufort were discarded^42,43^; and 3) kernel density estimation with reference bandwidth selection was used to eliminate spatial outliers using the 95% area contour^44^.

### Environmental data

Environmental variables used in the analysis were expected to influence the distribution of loggerheads (e.g.^45^). In addition to the four environmental factors that were recorded by the observers at the time of observation (Beaufort sea state, glare, visibility and the time of day), we included eight environmental factors: depth, seabed slope, distances to the coast (DcoastKm), to the 500 m (D500Km) and to the 1000 m (D1000Km) isobaths, sea surface temperature (SST), net primary productivity (NPP) and sea surface height deviation (SSHd). Depth was available at a 0.0027 degree grid cell resolution and was derived from acoustic bathymetric data analyzed in Vasquez *et al*.^46^. Slope, DcoastKm, D500Km and D1000Km were calculated from these bathymetric data using ESRI ArcGIS 10.1, and were included to indicate proximity to topographic features like seamounts and oceanic islands. Remote sensing data products (SST, NPP and SSHd) were obtained from NOAA’s Coast Watch program (http://coastwatch.pfeg.noaa.gov/). Daily SST was obtained at 0.25 degree grid cell resolution from the Reynolds Optimum Interpolation V2 high-resolution blended analyses, which used data from the NOAA National Climatic Data Center (NCDC) Advanced Very High Resolution Radiometer (AVHRR). Monthly averaged NPP (in mg C m-2 day-1) was obtained at 0.1 degree grid cell resolution, and was calculated from Chlorophyll-a concentration and photo-synthetically available radiation (PAR) measured by the SeaWiFS sensor and SST measurements from the NOAA Pathfinder Project. Daily SSHd was a merged product from various altimetric missions (Topex/Poseidon, ERS-1/2, Geosat Follow-On, Envisat and Jason-1) and was provided by Aviso at a 0.25 degree grid cell resolution. SSHd was included to indicate meso-scale phenomena such as eddies. Loggerhead sea turtle counts were spatially allocated to the average geographic position of each transect, and intersected with environmental data. Besides these environmental variables we also included latitude and longitude in the models to account for unexplained spatial variance. To evaluate the effect of time of day, we created a categorical variable in which the six daily periods were divided into three groups (09:00 and 11:00: morning; 13:00 and 15:00: midday and 17:00 and 19:00: afternoon).

### Probability of detection

The detection probability of loggerhead sea turtles during transects was estimated using conventional (CDS) and multiple-covariate distance sampling (MCDS)^47–49^.

After a preliminary analysis of the distance data, smearing was applied to the recorded sighting angles and distances to account for rounding errors resulting from the visual estimation, so called heaping^47,48^. Uniform smearing was applied over the sector defined by the angle range (Ө − ф, Ө + ф) and distance range (r*(1 − s), r*(1 + s)), where Ө and r are the measured angle and distance to the sighting, ф is the smearing angle and s is the proportional sector of distance to use as the basis for smearing. Since the observed angle and distance distributions showed marked spikes at 10-degree and 10-meter intervals respectively, smear parameters were set to ф = 5° angle and s = 0.2 m distance. Perpendicular distance of the sightings to the transect line was calculated as d = r*sin θ^47^. Data truncation was applied at 100 m in order to eliminate outliers and improve model fitting^48^, resulting in a strip width of 200 m.

Hazard-rate and half-normal detection functions with null, cosine and polynomial adjustments were fitted to the perpendicular distance data (n = 429 detections) grouped into distance bins (0–5, 5–10, 10–20, 20–30, 30–40, 40–50 and 50–100 m), along with covariates that could influence the detection of turtles (Beaufort sea state, glare and vessel speed). Best model fit was selected using Akaike’s Information Criterion (AIC) and the final model fit was assessed using diagnostic plots and goodness-of-fit chi-square test^50^. All analyses were performed using the “Distance” package in R^51^.

### Data analysis

Generalized Additive Mixed Models (GAMMs) were used to relate the density of loggerheads with topographic, environmental and spatially explicit variables in order to derive an unbiased index of relative abundance for the study area between 2001 and 2015. Such approach is particularly useful for surveys from opportunistic platforms, when the survey design cannot be randomised^52,53^. The density of loggerhead sea turtles was modelled using the number of loggerheads sighted as the response variable and the logarithm of the effective area surveyed (i.e. transect length x strip width x sighting probability) as offset term^53,54^. Candidate explanatory variables consisted of the year, vessel speed and the environmental variables. The observer ID was included as a random effect to account for any observer effect, whereas inclusion of vessel ID did not improve the models. Sightings were modelled as count data assuming a zero-inflated Poisson distribution (ZIP-GAMM), because Poisson and negative binomial distributions were shown to impose incorrect assumptions on the data. Data examination revealed a high proportion of zero counts (98%, n = 17419) and the distribution was over-dispersed (i.e. variance > mean) compared to the traditional Poisson distribution (i.e. variance = mean). ZIP models were considered appropriate for the data, because a ZIP distribution allows for over-dispersion and calculates relative abundance as a mixed process with binary and Poisson distributions^55^. Through model selection, one-stage zero-inflated Poisson models (ziP link function in mgcv^56^) were shown to outperform two-stage models (ziplss link function in mgcv^56^). The GAMM models were built by backward selection of individual smooth terms and tensor product smooths. Model selection was based on information criteria (AIC) because model parameters were fitted using penalized likelihood maximization (gam function from the mgcv package^56^). To reduce chances of overfitting, the number of knots of the smoothers were adjusted whenever appropriate. Nested models were compared using significance testing (Chi-square test). Model assumptions were evaluated through the inspection of diagnostic plots. Annual estimates of relative abundance were derived from the model estimates for each year. All analyses were performed in R^57^.

Nonparametric bootstrap (“boot” package in R^58^) was used to estimate the true variance in the annual estimates of relative abundance obtained from the models^53,54^. Both the detection function and GAMM were refitted for each of 1000 new datasets which were resampled from the original data, with replacement and independently for each year. The bootstrap standardised errors (BSE) of the annual estimates are reported, while the 2.5^th^ and 97.5^th^ percentiles were used as the 95% confidence intervals (BCI), making no assumptions about the underlying distribution^53,54^.

To explore the relationship between model estimates of relative abundance for the Azores and nest counts from Florida core index beaches (FCIB, data courtesy of S. Ceriani, Florida Fish and Wildlife Conservation Commission and Fish and Wildlife Research Institute Index Nesting Beach Survey Program Database as of 25 Sept. 2016) the model derived annual estimates of relative abundance were lag-plotted against annual nests counts for 0- to 12-year lags and the cross-correlation calculated using the *astsa* package for R^59^.

## Results

### Data filtering

After filtering, the dataset consisted of 17,761 transects covering 67,992 km within a 95% kernel core area of 198,401 km^2^. This dataset contained sightings of 429 loggerheads that were recorded along 393 transects (Fig. 1). Details on the number of transects and sightings retained for analysis per filtering step and per year are summarized in supplementary material (Table S1). The mean annual number of transects included in the analysis over the 15 yr. was 1184.1 (SD ± 361.5) for a mean annual effort of 4528.14 km (SD ± 1338.02) and mean transect length of 3.82 km (SD ± 0.87). The dataset further showed some considerable variability in the number and spatial distribution of transects in relation to the different years and months (supplementary material Figs S1–S2).

### Probability of detection

**Figure 2:**
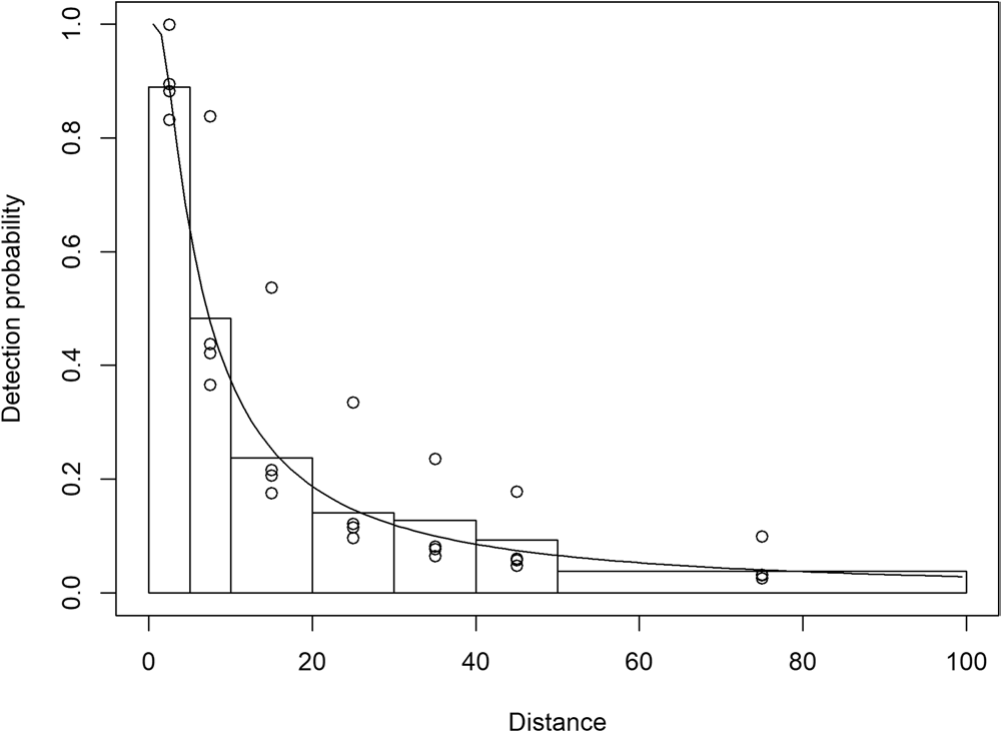
Observed distances and fitted detection function for loggerhead sea turtles during visual line transects performed between 2001 and 2015 by fishery observers from the Azores Fishery Observer Program (POPA - Programa de Observação das Pescas dos Açores) onboard pole-and-line tuna vessels. The line shows the average detection function, while the points represent the estimates in function of the Beaufort sea state.

The detection function with lowest AIC was the hazard-rate function with null adjustment and with Beaufort sea state as a covariate. The average probability of detection per transect was 0.148 (±0.020 SE) and varied between 0.113 (Beaufort 2) and 0.274 (Beaufort 0) according to Beaufort sea state (Fig. 2). The chi-square value of the goodness of fit test was marginally significant (p = 0.03) owing to a lack of observations between 5 and 10 m, but was not deemed sufficient to prevent using the model for inference.

### Data analysis

**Figure 3:**
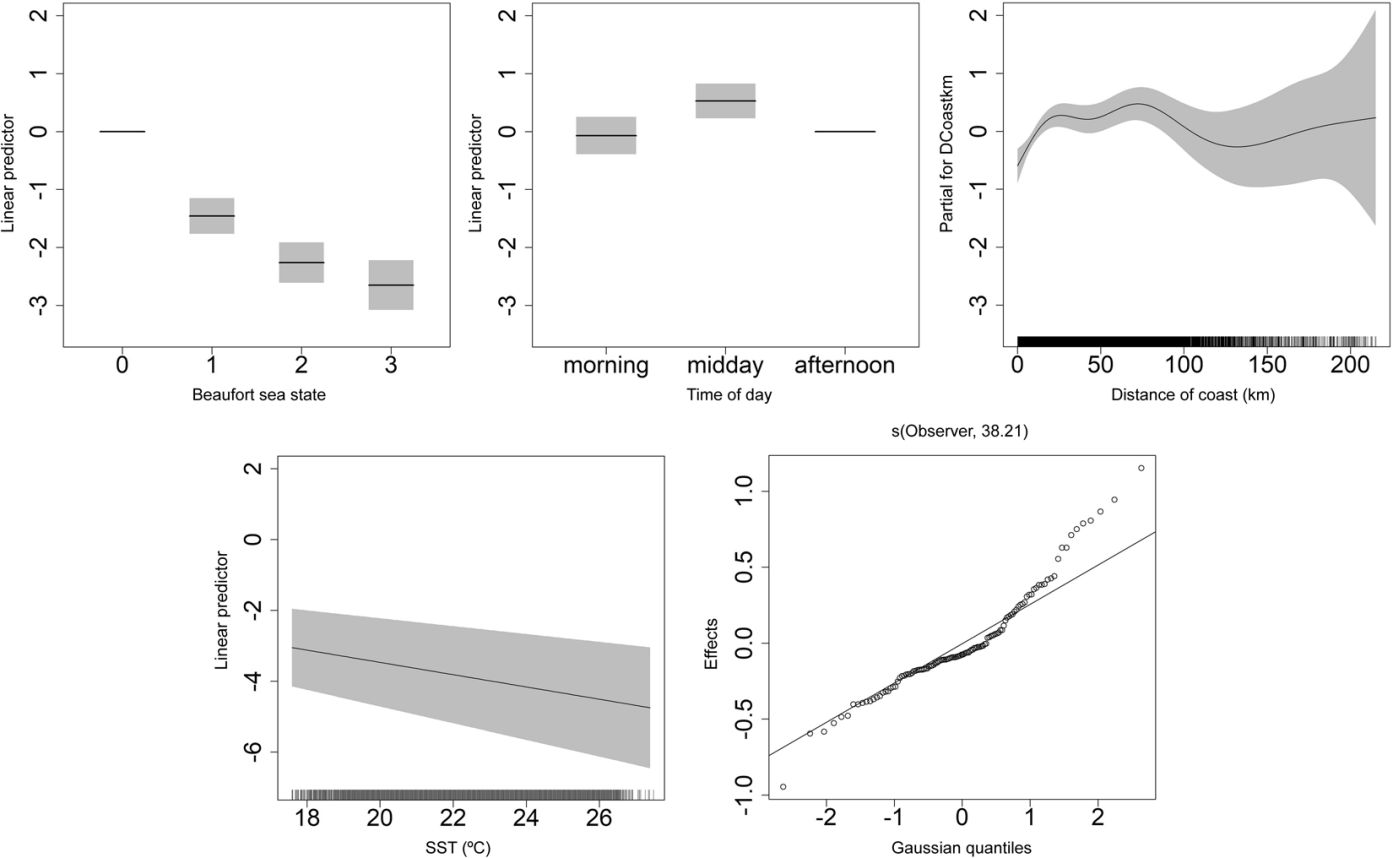
GAMM generated partial effects of Beaufort sea state (top left), Time of day (top center), distance from the coast (top right) and SST (bottom left) on the density of loggerhead sea turtles in the Azores calculated from the POPA visual sightings database (2001–2015). The QQ-plot (bottom right) shows the Gaussian quantiles of the observer effect.

**Figure 4:**
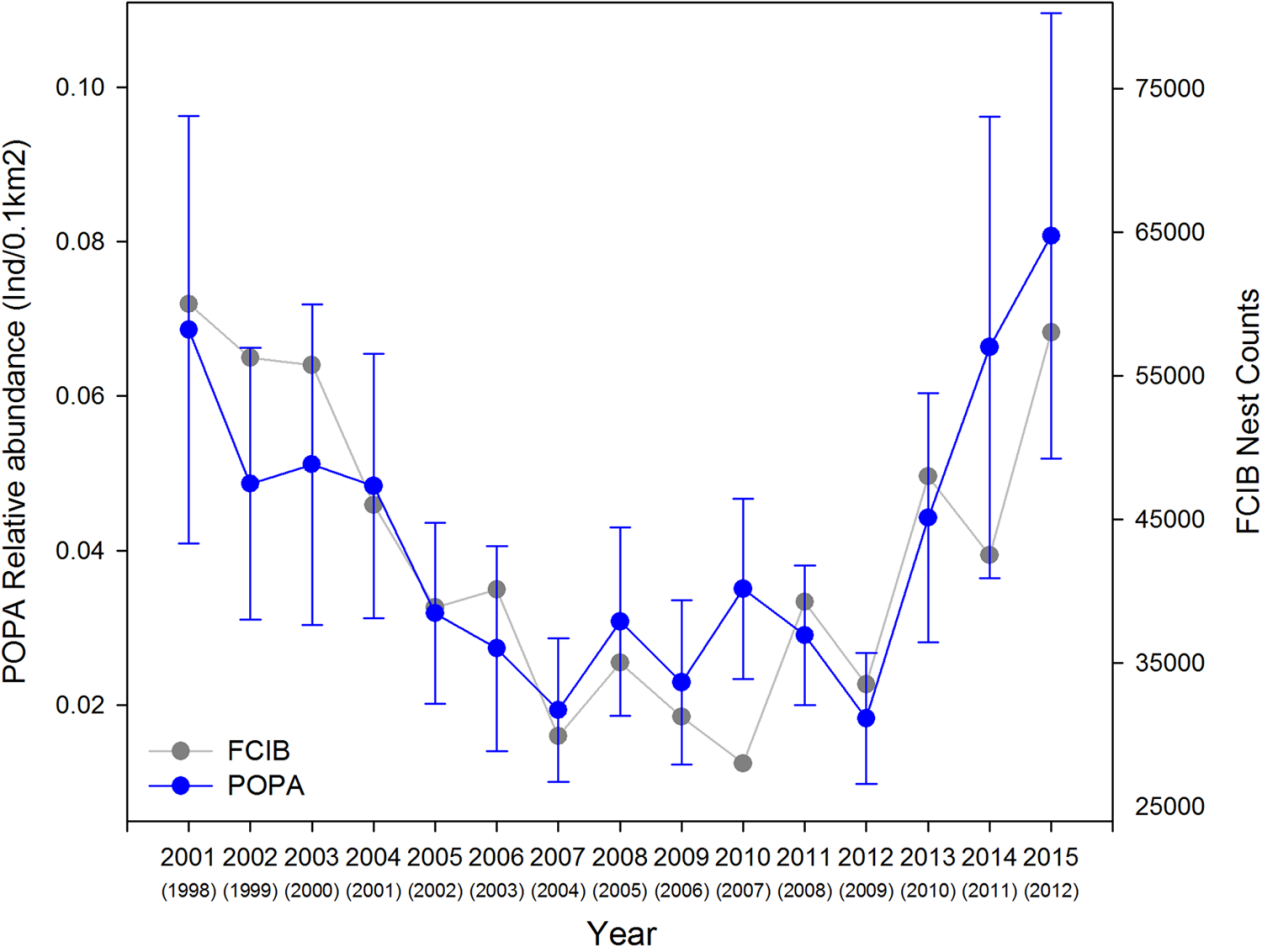
GAMM derived annual indices of relative abundance (Ind./0.1 km^2^; ±BSE) of loggerhead sea turtles in the Azores calculated from the POPA visual sightings database (2001–2015), compared with annual nest counts from Florida core index beaches (Index Nesting Beach Survey - Florida Fish and Wildlife Conservation Commission; 1998–2012). The X-axis shows the year of the POPA sightings, matched with the year from the annual nest counts from Florida core index beaches in parentheses, assuming a 3-year lag. Blue – POPA visual sightings; Grey - annual nest counts from Florida core index beaches.

The results of the final ZIP-GAMM are summarized in Table 1. The independent variables that were retained after model selection and validation were year, time of day, sea state, SST and distance to the coast. The model explained approximately 75.6% of the variability. Plots of the partial effects of the different covariates are included in Figs 3 and 4. The model showed a strong effect of sea conditions on the observed density of loggerhead sea turtles, i.e. an increase with decreasing sea state measured in Beaufort. The effect of SST displays a strong negative trend over the range from 18° to 25 °C. The relationship with distance to coast indicates a lower density of sea turtles up to approximately 10 km from the coast line. For the influence of time of day, the model shows higher values during midday relatively to morning and afternoon periods.

**Table 1:** Results of the GAMM model for visual sightings of loggerhead sea turtles from pole-and-line tuna vessels in the Azores.

	Estimate	Std. Error	*z* value	*p*-value
(Intercept)	1.95	0.76	2.57	0.01
	**df/edf**	**Ref.df**	**Chi.sq**	***p*** **-value**
factor(Year)	14		36.52	8.71E-04
factor(Beaufort)	3		46.48	4.49E-10
factor(Time group)	2		23.12	9.56E-06
SST	1		27.19	1.84E-07
s(Observer,bs = “re”)	39.28	119	82.16	3.94E-08
s(DcoastKm)	5.61	6.75	30.24	6.79E-05
**Deviance Explained** = 75.6%

**Figure 5:**
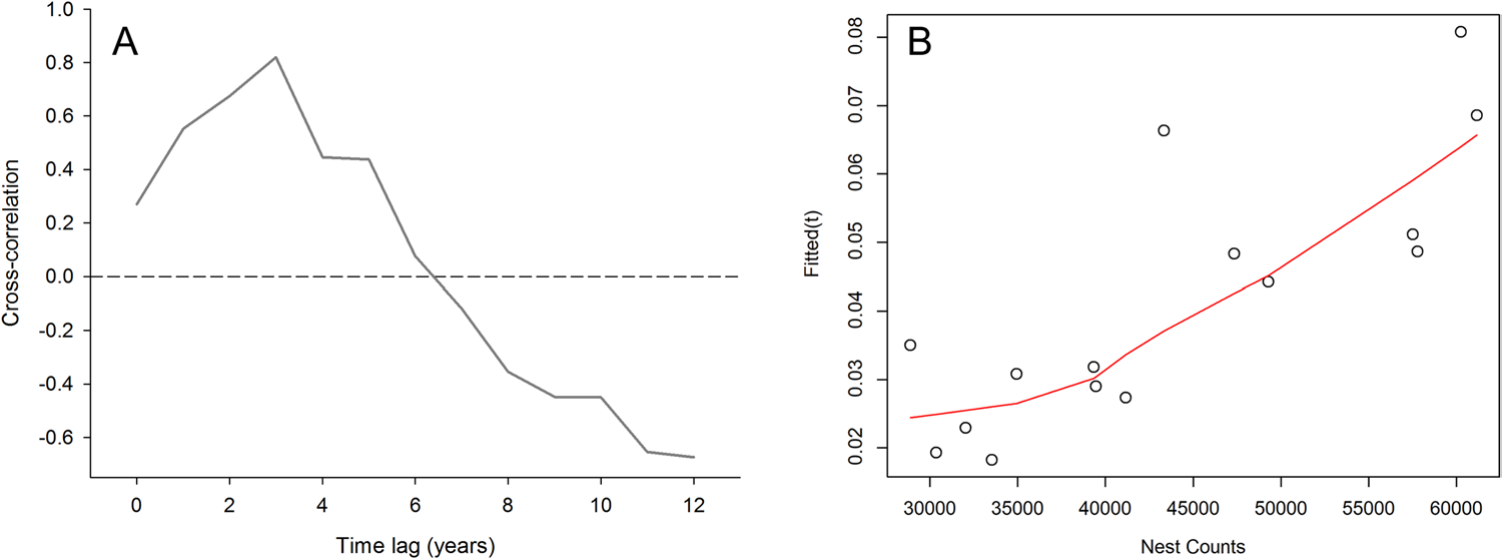
Panel A: Cross-correlation between the GAMM derived annual relative abundance of loggerhead sea turtles in the Azores calculated from the POPA visual sightings database (2001–2015), and annual nest counts from Florida core index beaches (Index Nesting Beach Survey - Florida Fish and Wildlife Conservation Commission; 1989–2015) for 0 to 12 year lags. Panel B: Lagged scatter plot for a 3-year lag (astsa package in R) of the GAMM derived annual index of relative abundance of loggerhead sea turtles in the Azores (2001–2015), versus annual nest counts from Florida core index beaches (1998–2012).

The analysis further revealed a significant effect of year on the relative abundance of loggerheads (Fig. 4). The relative abundance of loggerhead sea turtles in the Azores region declined 67% (18%–84% BCI) from 0.0689 Ind./0.1km^2^ (±0.0277 BSE) in 2001 to 0.0229 Ind./0.1km^2^ (±0.0106 BSE) in 2009. From 2012 to 2015 the relative abundance increased 342% (87%–801% BCI) to 0.0808 Ind./0.1km^2^ (±0.0289 BSE). This pattern showed a very close correspondence when lag plotted against the annual nest counts from Florida core index beaches (Fig. 4). Lag-analysis showed the highest cross-correlation (r = 0.82) for a 3-year lag between nest counts and the modelled relative abundance based on sightings from Azorean tuna vessels (Fig. 5; Supplementary material Fig. S3). While the lagged relative abundance in the Azores generally tracked annual nest counts very closely (e.g. 2008 and 2012), despite some evident departures such as for the years 2010 and 2014, it is important to note that such correlation does not establish a causal relationship.

## Discussion

This study provides the first assessment of relative abundance of oceanic-stage juvenile sea turtles. Based on 15 years of standardised visual transects performed on opportunistic platforms, this study provides important information for this key life stage for which little demographic information is currently available. Since Archie Carr’s landmark study on the “lost year(s)”^32^, the importance of the Azores for the Northwest Atlantic loggerhead population has been extensively studied and documented^9,13,33,36–38,60^. However, this is the first study to establish a quantitative link in addition to the already established genetic link^33,34^ between oceanic-stage juvenile loggerheads in the archipelago and source rookeries 5000 km away.

The model revealed the existence of a strong effect of the year on the relative abundance of loggerhead sea turtles in the study area and a close correspondence with the pattern of nest counts in source rookeries in Florida. Nest counts recorded on Florida core index beaches displayed a dramatic decline by 43% between 1998 and 2006 and have been recovering since^61^. Concurrently, relative abundance of oceanic juvenile stage loggerheads displayed a lagged response with a 67% (18%–84% BCI) decrease and subsequent increase. Besides this general trend, it appears that even smaller oscillations in relative abundance (e.g. 2008 and 2012) were predictable based on nest counts from corresponding years. We therefore conclude that the relative abundance of oceanic juvenile stage loggerheads was largely driven by the production of nests in the source rookeries and that recruitment was largely stable during the studied period.

The effect of climate forcing on demographic processes in sea turtles has received considerable emphasis, in particular its influence on sex determination^62,63^, nest distribution^64^, nesting activity^65–67^ and growth rates^68–70^. A possible link between declining annual nest counts of loggerheads in the Northwest Atlantic and the observed environmental regime shift, which was responsible for declining growth rates in three Atlantic sea turtle species^70^, has also been hypothesised and still requires further evaluation^68^. Alternatively, recent modelling studies have proposed a strong effect of historical climate forcing on neonate survival, mediated through oceanic current dynamics, to explain trends in annual nest counts^30,31^. However, the latter mechanism implies a highly variable neonate survival^30,31^ and consequently a variable recruitment to the open ocean, which would be expected to offset the observed correlation between nest abundance and the relative abundance in the open ocean. Hence, it appears unlikely that population growth has depended decisively on variable, climate-mediated survival during the first year of life^30,31^. Our study thus provides empirical evidence for the key assumption of minimal variability in survival rates during the early oceanic stage which supports contemporary conservation planning for Northwest Atlantic loggerhead sea turtles.

Overall, stochastic effects appear to be less important than would be expected based on their influence on individual processes such as nest destruction, hatching and emergence success, and dispersion by ocean currents^71–75^. Variable recruitment has been extensively documented for marine invertebrates and fish species with productive, r-selected life histories^76–80^, as opposed to loggerhead sea turtles, which are long-lived, late maturing organisms with low fecundity^29^. Although neonates are largely inactive, low energy, float-and-wait foragers^9,43,81^, potential behavioural plasticity between active and passive behaviours in early juveniles may further contribute to their resilience against environmental fluctuations^9,82^.

The 3-year lag between nest counts and abundance estimates in the Azores corresponds approximately to the age of loggerheads with 25 cm CCL^12,36^, which is in line with the observed detection size of sea turtles from Azorean tuna vessels^13,60^. The reported age distribution assessed between 1990 and 1992 in the Azores showed that approximately 50% (between 43% and 60% for individual years) of the individuals sampled on pole-and-line tuna vessels were between 2 and 4 years of age^13^, so that a 3-year lag seems justified. Nevertheless, further analysis and contemporary characterization of the demography in terms of age distribution and origin will be necessary to fully reconcile the presence of multiple age classes in the Azores and the close correspondence with nest counts from Florida core index beaches.

Our model was able to explain a large proportion (75.6%) of the variability in the sighting data. Since the effect of sea conditions on the detection probability was adjusted for in the detection function, the environmental variables that were retained were likely either linked to availability (time of day, Beaufort sea state and SST) and habitat characteristics (SST and distance to coast). Availability for detection depends on the time sea turtles spend at the surface, and is likely influenced by the time of day and the sea state. Surface behaviour of oceanic-stage loggerheads satellite tagged off Madeira was shown to vary seasonally, was more frequent during the day, particularly during spring and summer, and was positively associated to conditions favouring absorption of solar radiation, such as low wind and elevated air temperature^83^. The decreasing number of observed loggerheads with SST higher than 18 °C is consistent with satellite tracking data in the North Pacific^84^. This negative trend in function of SST in combination with its range and low resolution suggests a seasonal effect, with a higher observed density during spring compared to summer. While this seasonal pattern can be related to availability bias^83^, it also challenges the concept of a closed population during the tuna fishing season. Juvenile loggerheads are known to display wide ranging movements^83,85,86^, including in the study area^87^, but seasonal dynamics remain poorly understood and will require further investigation.

Some variables that were expected to influence the relative abundance were not retained during model procedures (e.g., latitude and longitude), or could not be included because of issues with collinearity (e.g., NPP). The spatial and temporal scales considered in the present study are an important factor and may not coincide with those on which we expect certain environmental variables and drivers to act (e.g., SSHd as proxy for meso-scale features^45,84,85,87^). For example, the variables that served as proxy for the presence of seamounts (i.e. slope, distance to the 500 m and 1000 m isobaths) were not retained, notwithstanding previously reported associations^39^. One of the reasons may be the high density of seamounts in the area in combination with their different levels of attraction to oceanic visitors and the variable scale of their influence radius^88^, which are likely to obscure their effects. Similarly, the effect of nest counts on the relative abundance is more likely to be detected when nest productivity itself undergoes significant change, as is the case for the Northwest Atlantic population. Longer time series are undoubtedly necessary to fully understand regulatory mechanisms and the effect of long-term climate forcing on recruitment.

Even though departures from the relationship with nest counts (e.g., 2010 and 2014) may be simply due to a lack of accuracy of the estimates, alternative explanations may relate to a low number of transects (e.g. 2014), variation in fisheries operations (e.g. 2010 and 2014), and/or possible distributional range shifts due to climate forcing (e.g. 2010). The years 2010 and 2014 were anomalous in terms of the tuna fisheries in the region. The year 2010 was characterized by an exceptional capture of skipjack tuna, by far the highest in the last 12 years, and an extension of the fishing season until November, whereas an average season ends towards the end of September^89^. In 2014, tuna landings in the Azores were the lowest in the last decade and consequently vessels operated mostly outside the Azores EEZ. In addition, the scarcity of tuna influenced boat captains to adapt their fishing operations (e.g., deep hand-lining with live bait and FAD-style concentration of fish under the vessel). Together, these operational changes resulted in a much lower number of visual transects (~−40%). The winter of 2009/2010 was also characterized by an exceptionally negative phase of the North Atlantic Oscillation (NAO), which was responsible for anomalous low SSTs in the central North Atlantic that were noticeable for several months^90–92^. Given the strong influence of SST on loggerhead distribution and behaviour (e.g.^93–96^), it is likely that the higher than expected relative abundance was also linked to some climate driven shift in distributional range or habitat compression^80,97^, as reported for pelagic fish such as sardines and anchovies^98^.

The present study corroborates the adequacy of standard visual sightings from opportunistic platforms as a useful tool for assessing the relative abundance of juvenile oceanic stage loggerhead sea turtles. The method allows for a sufficiently high sighting effort necessary to assess this low density life stage. This is particularly relevant due to the importance of sea state variables on detectability and availability, since suitable sea conditions can be a limitation. The filtered dataset represents a cumulative 4440.25 hours of visual sighting effort, or 296.02 hours/yr (SD ± 90.38). A dedicated annual survey with comparable effort, seasonal and spatial coverage is usually not feasible due to financial and logistical constraints. Simultaneous data collection on multiple platforms by fisheries observers represents a valid alternative as it allows sharing resources with fishery observer programs, which are often mandatory, and achieving a broad spatial coverage. In contrast, its dependence on fishing activity can be a limitation for consistent and long term monitoring, when fishing operations or fishery monitoring obligations change.

Continued monitoring of oceanic juvenile stage loggerheads is critical. Current assessment of loggerhead sea turtle populations relies too heavily on abundance estimates of nesting females^10^. Our study demonstrates that monitoring the oceanic juvenile stage can provide an additional assessment stage in the long life cycle of loggerheads, allowing the detection of population disturbances during the first years of life (“early warning system”), either from anthropogenic or natural causes. Further improvements of the assessment can be expected through the generation of credible estimates of absolute abundance for the area^47,48^. This progress depends on the verification of the method’s assumptions and the quantification of availability bias, which is linked to surface behaviour and mediated by environmental variables^99^. Obtaining reasonable and reliable absolute abundance estimates would be an important step, as it would allow a more thorough interpretation of mortality from by-catch or other anthropogenic threats.

## Supplementary information

Supplementary material

## Data Availability

Sea turtle sighting data from the Fisheries Observer Program of the Azores POPA^41^ are accessible through the OBIS (http://www.iobis.org/) and EMODnet (http://www.emodnet-biology.eu/) open access repositories.
