# Supplementary material for: Relative abundance of oceanic juvenile loggerhead sea turtles in relation to nest production at source rookeries: implications for recruitment dynamics
Source: Sci Rep. 2019 Sep 10;9:13019. doi: 10.1038/s41598-019-49434-0 (PMC6737082; doi:10.1038/s41598-019-49434-0)
Supplement: Supplementary file 1 — Supplementary material [file 41598_2019_49434_MOESM1_ESM.doc]

# Electronic Supplementary Material

**Relative abundance of oceanic juvenile loggerhead sea turtles related to nest production at source rookeries: implications for recruitment dynamics**

**Frederic Vandeperre^1,2,3*^, Hugo Parra^1,2,3^, Christopher K. Pham^1,2,3^, Miguel Machete^1,2,3^, Marco Santos^4^, Karen A. Bjorndal^5^ and Alan B. Bolten^5^**

1 - IMAR – Institute of Marine Research, Departamento de Oceanografia e Pescas, Universidade dos Açores, Horta, Portugal

2 - MARE – Marine and Environmental Sciences Centre, Departamento de Oceanografia e Pescas, Universidade dos Açores, Horta, Portugal

3 - OKEANOS – Departamento de Oceanografia e Pescas, Universidade dos Açores, Horta, Portugal

4 - DRAM – Regional Directorate for Sea Affairs, Colónia Alemã, Horta, Portugal

5 - ACCSTR – Archie Carr Center for Sea Turtle Research, University of Florida, Gainesville, FL, USA

**Table of Contents**

**Table S1** - Details on the number of sightings (Nind) and transects (Ntransects) retained at each step of the filtering procedure for each year of the POPA visual sightings database (2001-2015) (Filtering procedure step1: transects with lengths ranging between 1.35 and 5.40 km; step 2: transects conducted with sea conditions below 3 Beaufort and; step 3: transects within the 95% kernel area contour).

**
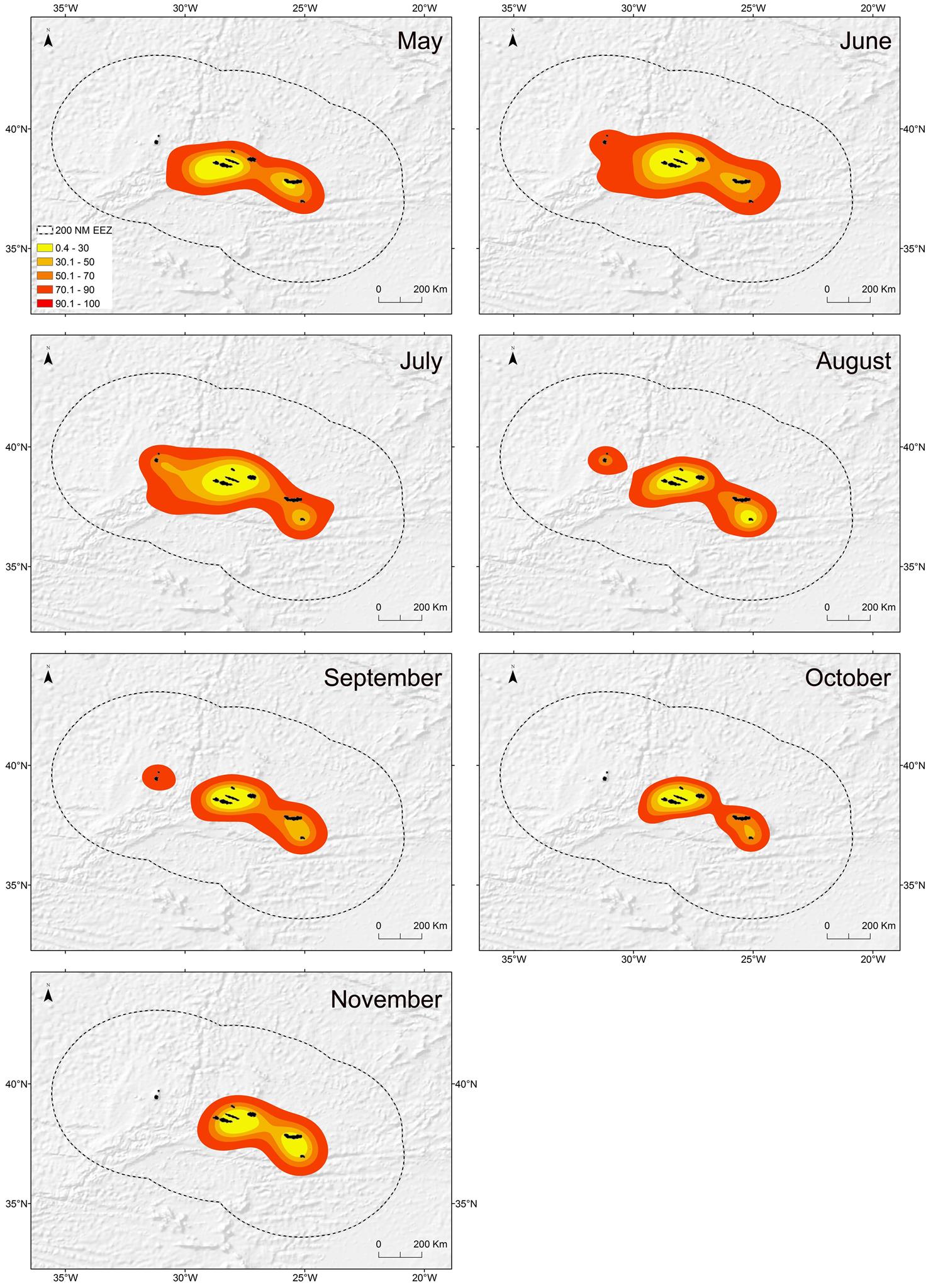
**

**Figure S1** – Kernel density surface showing the monthly distribution of visual transects from the POPA visual sightings database (2001-2015) after the data filtering procedure.

**
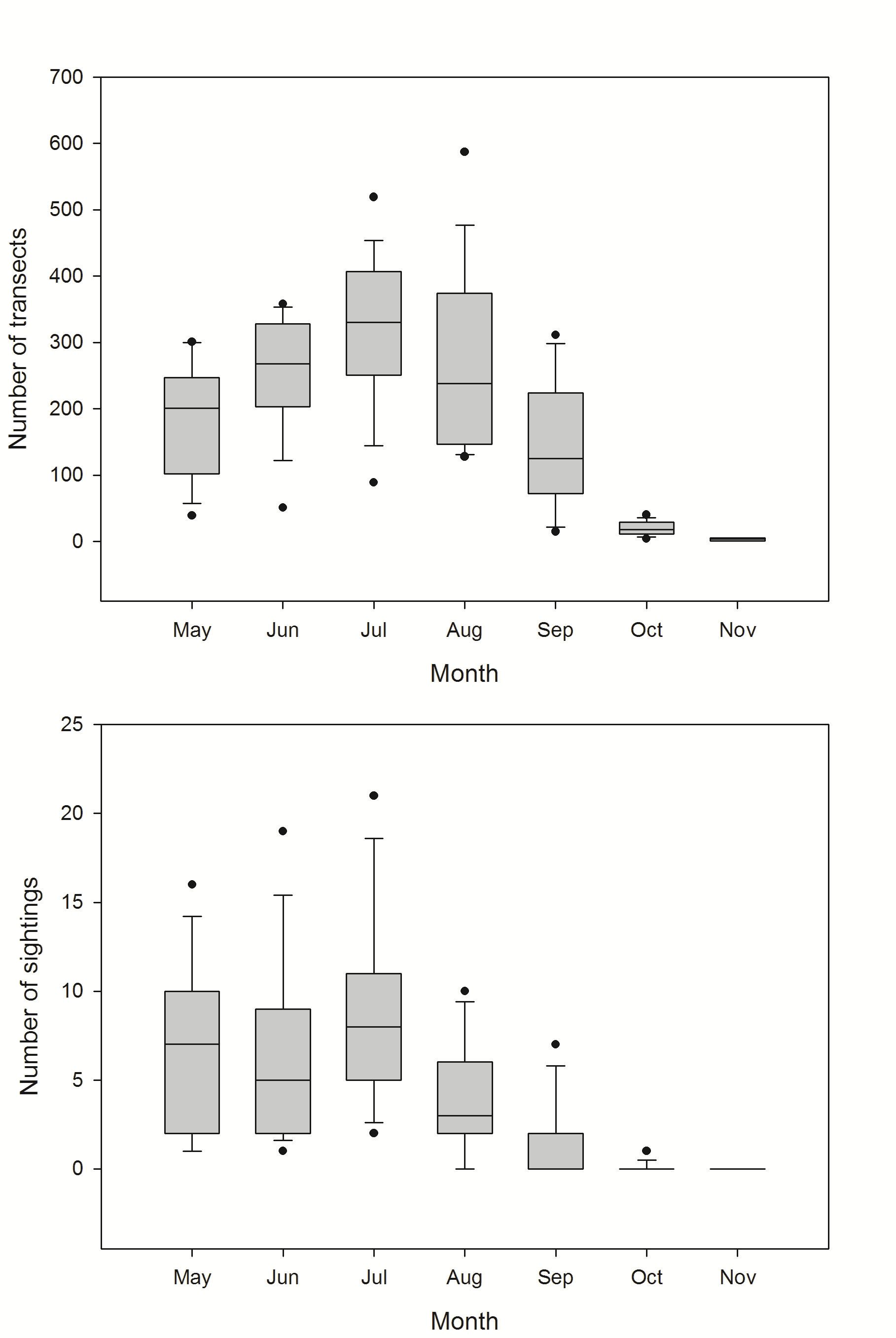
**

**Figure S2** – Boxplot showing the monthly number of transects and sightings from the POPA visual sightings database (2001-2015) after the data filtering procedure.

**
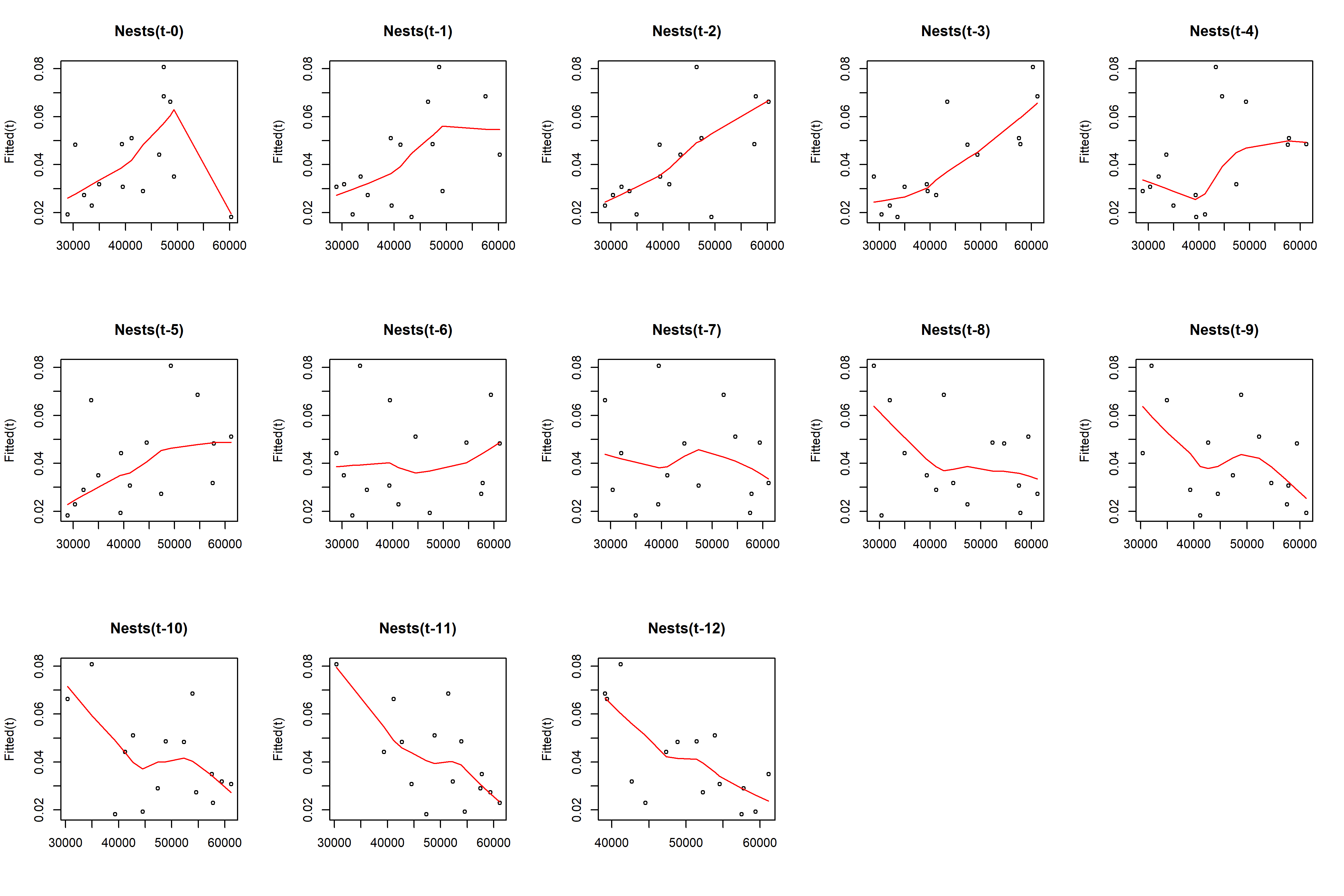
**

**Figure S3** – Lagged scatter plots of the GAMM derived annual relative abundance of loggerhead sea turtles in the Azores calculated from the POPA visual sightings database (2001-2015), versus annual nest counts from Florida core index beaches (Index Nesting Beach Survey - Florida Fish and Wildlife Conservation Commission; 1998-2012) for 0 to 12 year lags (astsa package in R).
